# Community mining of open clinical trial data

**DOI:** 10.18632/oncotarget.20853

**Published:** 2017-09-13

**Authors:** Teemu D. Laajala, Justin Guinney, James C. Costello

**Affiliations:** James C. Costello: Department of Pharmacology, University of Colorado, Anschutz Medical Campus, Aurora, CO, USA

**Keywords:** data sharing, metastatic castration-resistant prostate cancer, survival analysis, machine learning, DREAM Challenge

There has been increasing attention given to how biomedical research is being conducted, with team science emerging as an alternative to more traditional, individual research laboratory models. In principle, it is generally accepted that working together as a community to share data, address questions, and leverage the collective knowledge is the optimal approach to biomedical research; however, there are many practical considerations that make community research difficult, particularly related to clinical trial data. Clinical trials are resource intensive and expensive to run, and the academic model of tenure and advancement dis-incentivizes the broad sharing of such data. Additionally, concerns about patient confidentiality can make it difficult for institutions to openly share data [1]. Despite these challenges, there are groups with the goal of driving biomedical research forward as a community.

The DREAM Challenges (http://dreamchallenges.org/), Sage Bionetworks (http://sagebase.org/), and *Project Data Sphere, LLC* (PDS, http://projectdatasphere.org/) are examples of the groups that aim to democratize data and build research communities around important biomedical questions. To this end, these same groups organized and led the Prostate Cancer DREAM Challenge (PCDC) with the goal of addressing the following two questions: 1) can we develop better models for predicting overall survival (OS) for patients with metastatic, castration-resistant prostate cancer (mCRPC) [2], and 2) can we develop better models to predict which patients are likely to discontinue Docetaxel treatment due to adverse treatment events [3]? Here, we describe the results for addressing the first question.

The PCDC was an open-data, open-community initiative that utilized archived data from the comparator arms of 4 phase III clinical trials. Data were compiled, cleaned and made available for participants to address the Challenge questions. Hundreds of international participants formed teams, developed models independently, and submitted predictions to Challenge organizers for objective evaluation and automated scoring. Reported in Guinney et al.[2], results from 50 international teams provide a robust benchmark of models to predict OS in patients with mCRPC. Over half of the submitted models significantly outperformed a benchmark model in the field [4]. The winning team - from the University of Turku and the Institute for Molecular Medicine Finland - developed a model based on an ensemble of penalised Cox regression models (ePCR). The model applied the ‘wisdom of the crowds’ principle inspired by previous DREAM Challenges [5–7], with each of its ensemble members capturing different key traits to the data. The performance of the ePCR approach can be attributed to combining multiple individual mathematical models into a stronger model and was supported by several rigorous modeling choices, namely the censoring (modeled by Cox regression), missingness (regression-based imputation), high amount of predictors (penalisation), and intrinsic structure of the data (unsupervised learning).

Post-hoc analysis of the 50 team predictions revealed consistent patterns in the patient populations with 3 distinct groups of high-, moderate-, and low-risk. When individual patient characteristics were assessed, known risk factors were confirmed to be predictive across teams and additional novel factors were identified. Aspartate aminotransferase was identified by half of the teams as having predictive value, likely reflecting dysregulated hepatic function. Other factors included total white blood cell count, absolute neutrophil count, red blood cell count, region of the world, body-mass index, and creatinine. Overall, the Challenge confirmed readily known risk factors, provided clinicians with improved novel tools for assessing patient health in mCRPC, and functions as a benchmark of methods for the field. Additional publications on predictive models for the PCDC can be found at:

http://f1000research.com/gateways/DREAMChallenges.

The results of the PCDC exemplify the benefits of providing clinical trial data to an open, collaborative and diverse community of data and biomedical scientists. Modern biomedical research is generating large amounts of data, and yet wide access to these data by the research community remains an ongoing challenge. Multidisciplinary collaborations among clinicians, data analysts, and biomedical scientists - exemplified by the PCDC - highlights the value for large-scale data sharing initiatives and the potential for accelerating the pace of biomedical discovery. The collective effort stemming from the PCDC demonstrated both that there is a large research community waiting to gain access to these data and that once these data and necessary infrastructure are made available, the research community can reach far beyond the capacity of single lab-driven research models.
